# Persistent Elevation of CA 19-9 Levels in the Long-term Follow-up before Laryngeal Cancer

**DOI:** 10.5005/jp-journals-10018-1222

**Published:** 2017-05-05

**Authors:** Hasan Özkan, Fatih Karakaya, Zihni Karaeren, Sibel Perçinel

**Affiliations:** 1 Department of Gastroenterology, School of Medicine, Ankara University, Ankara, Turkey; 2 Department of Biochemistry, School of Medicine, Ankara University, Turkey; 3 Department of Pathology, School of Medicine, Ankara University, Turkey

**Keywords:** CA 19-9, Laryngeal cancer, Severe reflux disease, Squamous cell carcinoma.

## Abstract

**Introduction:**

CA 19-9 is used as a tumor marker in colon, pancreas, biliary, and gastric cancers. Laryngeal cancer is the most common malignant epithelial tumor among head and neck cancers and has no specific tumor marker.

**Case report:**

A 66-year-old male patient had severe reflux symptoms during 5 years and had an isolated CA 19-9 elevation. Follow-up analysis revealed that he had larynx cancer and after laryngectomy, CA 19-9 levels decreased to normal range.

**Discussion:**

Currently, CA 19-9 is not a marker for malignancy. Laryngeal carcinoma has no specific tumor marker, but laryngeal squamous cell carcinoma may be manifested by elevated CA 19-9 levels.

**How to cite this article:** Özkan H, Karakaya F, Karaeren Z, Perçinel S. Persistent Elevation of CA 19-9 Levels in the Long-term Follow-up before Laryngeal Cancer. Euroasian J Hepato-Gastroenterol 2017;7(1):92-94.

## INTRODUCTION

CA 19-9, which is used as a tumor marker, was first developed in the 1980s as a monoclonal antibody against Sialyl-Lewis A antigen. It is known to be produced in normal cells (biliary, gastric, colonic, and pancreatic cells) and also to have an increased production when sialyl transferase enzyme is inhibited.^1^ CA 19-9 levels increase in colon, pancreas, biliary, and gastric cancers as well as conditions other than malignancies, such as cholangitis. It is usually used for the diagnosis of pancreatobiliary and gastrointestinal cancers, and especially as a combination with radiological methods to be helpful for follow-up after treatment.^2,3^ Laryngeal cancer is the most common malignant epithelial tumor among head and neck cancers. The most important risk factor is smoking and alcohol consumption, and the risk improves substantially when both factors are combined. The condition is more common in patients above 50 years of age and it is also more frequently seen in males. Over 95% of laryngeal cancers are squamous cell carcinomas. Histologically, they can rarely be adenocarcinomas, sarcomas, and neu-roendocrine tumors. There are no specific tumor markers for the prognosis and follow-up of laryngeal cancer.^4^

## CASE REPORT

**Graph 1:**
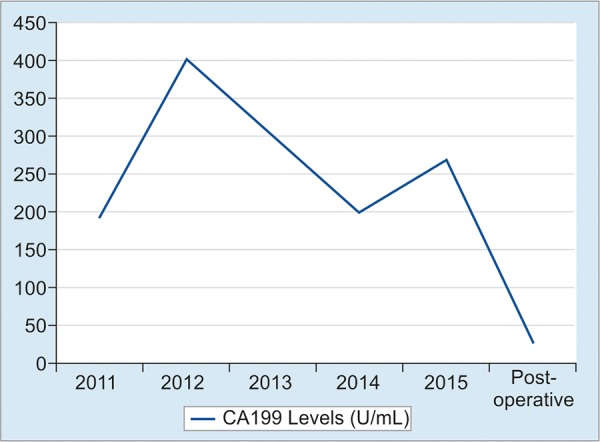
Values of CA 19-9

**Fig. 1:**
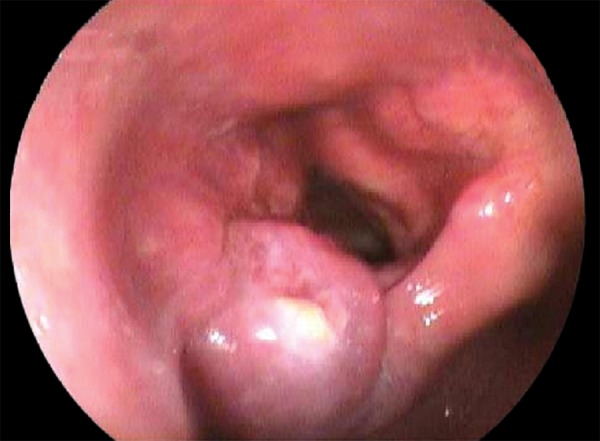
Endoscopic view of larynx cancer

A 66-year-old male patient was admitted to our institution after he underwent follow-up in another institution. CA 19-9 levels were found to be elevated. He had no known diseases other than hypertension. There were no alarming symptoms in his systemic examination, such as weight loss, abdominal pain, hematemesis, and night sweats. He had no history of alcohol consumption and abstained from smoking for last 20 years, after smoking for 20 years. The patient was found to have chronic reflux symptoms, which did not regress with proton pump inhibitors. The patient had undergone a gastrectomy and a billroth 1 anastomosis long time ago. No pathological findings were observed in the initial laboratory tests; both biochemical parameters and total blood counts were normal, including hemoglobin, which was found to be 16.6 gm/dL. The tumor marker tests were renewed and CA 19-9 was 193 U/mL, carcinoembryonic antigen was 2 ng/mL, alpha-fetoprotein was 4 ng/mL, and CA 72-4 was 1.1 U/mL, which showed that the patient had an isolated CA 19-9 elevation. To rule out a possible malignity concerning the gastrointestinal system, a gastrointestinal system endoscopy and colonoscopy were performed. Endoscopic and colonoscopic examinations had no findings pointing to a malignity. A former gastric operation scar was observed at the upper gastrointestinal endoscopy and the lower esophageal sphincter was found to be relaxed, explaining the reflux symptoms. To rule out a possible pancreatobiliary system malignity, abdominal computerized tomography and magnetic resonance cholangiopancreatography were performed, but no findings related to a malignity that can be at the pancreas or the biliary system were observed in the radiological imaging. As the patient had persistent CA 19-9 elevation with no pathological findings to explain this situation, a follow-up was initiated. As the patient had severe reflux symptoms, during the next 5 years, three gastrointestinal endoscopies were performed. In the endoscopic examination, similar findings were observed with the prior endoscopy. Again, during the follow-up, as the CA 19-9 levels were not stable, another colonoscopic examination was performed, but no findings pointing to a malignity were observed. During this period, the patient was examined with an abdominal ultrasound once in every year but no malignity-related findings were observed. During these years, although CA 19-9 was always 5 to 15 times the normal value, no other tumor markers were found to be elevated (Graph 1). Due to the fact that the patient had dyspepsia and persistent reflux symptoms, an endoscopy was performed, which showed a mass lesion above the larynx near the vocal cords with a malignant-like appearance. The patient was consulted to surgical departments. Before the operation, positron emission tomography was performed, and at the mass, at the left aryepiglottic area, and at the nodule in the third level of the left side of the neck, pathological 18F-fluorodeoxyglucose uptakes were observed. The patient underwent a total laryngectomy and lymph node dissection. Histopathological diagnosis was consistent with a moderately differentiated squamous cell carcinoma. Immunohistochemically, focal strong CA 19-9 positivity was detected in the apical areas of the cytoplasm of the tumor cells (Fig. 1). After the surgery, CA 19-9 levels were back to normal.

## DISCUSSION

**Fig. 2:**
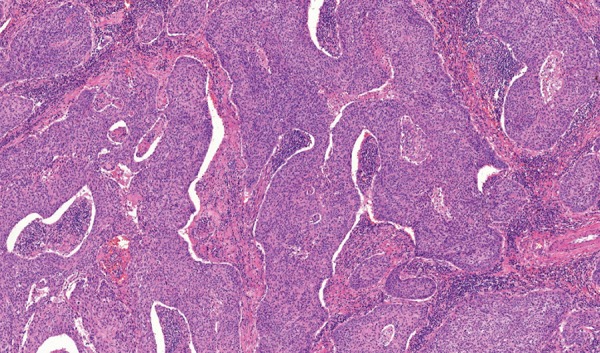
Moderately differentiated squamous cell carcinoma (H&E 7.1*)

**Fig. 3:**
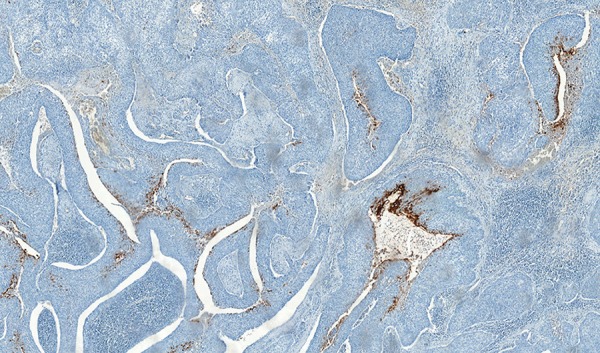
Focal strong CA 19-9 positivity in the tumor cells (diaminobenzidine 4.8*)

Generally, tumor markers are used to screen a possible disease relapse for patients with malignancy diagnosis. Tumor markers are not distinctly specific to a type of cancer and they are also produced in cells that belong to healthy individuals. As the tumor biology differs in every patient, tumor marker elevation does not result in the same way for each patient. The tumor markers may even be normal, even though the same cancer types are observed in the patients.^3^ CA 19-9, being produced as a monoclonal antibody against Sialyl-Lewis A antigen, apart from the known cancer localizations, is shown to do more color uptake in the squamous cell laryngeal carcinoma rather than normal larynx tissue (Fig. 2). CA 19-9 levels were also above the normal level when parts of larynx were edematous.^5^ In our case, the persistent CA 19-9 is thought to be the cause of the laryngeal mucosa being edematous as a result of severe reflux. Also, focal CA 19-9 expression detected immunohistochemically by the tumor cells and serum CA 19-9 level lowering to normal values show that the source of the tumor marker is the laryngeal tissue. In conclusion, even though CA 19-9 is not a specific tumor marker, i.e., put forward for laryngeal cancers, persistent CA 19-9 elevation should remind the clinician for laryngeal cancer during differential diagnosis, after gastrointestinal and pancreatobiliary cancers are ruled out (Fig. 3).

## CONCLUSION

We assume that a laryngeal squamous cell carcinoma may produce CA 19-9. Currently, CA 19-9 is not a specific marker for a malignancy so that it cannot be used for diagnosis of any tumor. But it should be kept in mind that laryngeal squamous cell carcinoma can be a reason of elevated CA 19-9 levels except gastrointestinal system tumors.
